# Structural Brain Alterations in Motor Subtypes of Parkinson’s Disease: Evidence from Probabilistic Tractography and Shape Analysis

**DOI:** 10.1371/journal.pone.0157743

**Published:** 2016-06-17

**Authors:** Griet Vervoort, Inge Leunissen, Michael Firbank, Elke Heremans, Evelien Nackaerts, Wim Vandenberghe, Alice Nieuwboer

**Affiliations:** 1 KU Leuven, Department of Rehabilitation Sciences, Tervuursevest 101/1501, 3001, Leuven, Belgium; 2 KU Leuven, Department of Kinesiology, Tervuursevest 101/1501, 3001, Leuven, Belgium; 3 Institute of Neuroscience and Newcastle University Institute for Ageing, Campus for Ageing and Vitality, Newcastle University, Newcastle upon Tyne, NE4 5PL, United Kingdom; 4 University Hospitals Leuven, Department of Neurology, Herestraat 49, 3000 Leuven, Belgium; KU Leuven, Department of Neurosciences, Herestraat 49, 3000, Leuven, Belgium; University of Tuebingen, GERMANY

## Abstract

**Background and Objectives:**

The postural instability and gait disorder (PIGD) and tremor dominant (TD) subtypes of Parkinson’s disease (PD) show different patterns of alterations in functional connectivity (FC) between specific brain regions. This study aimed to investigate the relation between symptomatic heterogeneity in PD and structural alterations underlying these FC changes.

**Methods:**

68 PD patients classified as PIGD (n = 41) or TD (n = 19) and 19 age-matched controls underwent Magnetic Resonance Imaging (MRI). Diffusion-weighted images were used to assess fractional anisotropy (FA) and mean diffusivity (MD) at the whole-brain level using tract-based spatial statistics (TBSS). In addition, structural connectivity was assessed between regions that previously showed altered FC using probabilistic tractography. Anatomical images were used to determine shape and volume of the putamen, caudate and pallidum.

**Results:**

TBSS revealed widespread FA reductions in PIGD compared to controls involving the superior longitudinal fasciculi and corpus callosum. No such differences were found in TD. Both PD subgroups had increased MD compared to controls in tracts connecting the left caudate with the bilateral ventral putamen. TD patients additionally showed increased MD compared to PIGD and controls in tracts connecting the right inferior parietal lobule with the right premotor and primary motor cortex, which previously showed altered FC. We also found grey matter atrophy in the rostrodorsal head of the caudate in PIGD compared to controls.

**Conclusion:**

Microstructural changes in white matter tracts, particularly in those connecting striatal sub-areas, partly underlie FC alterations in PD subtypes. Caudate shape alterations further implicate the striatum in PIGD pathophysiology.

## Introduction

Symptomatic heterogeneity in patients with Parkinson’s disease (PD) has led to the distinction between the postural instability and gait disorder (PIGD) and tremor dominant (TD) subtypes [1]. Although the classification procedure is solely based on gait, balance and tremor-related items of the Unified Parkinson’s Disease Rating Scale (UPDRS) [1, 2], accumulating evidence suggests that both subtypes are also characterized by different non-motor symptoms [3–6], altered distal motor control [7, 8] and distinct neural features [9–14]. Understanding the neural correlates at a functional and structural level using multimodal analysis is crucial to pinpoint the mechanisms leading to motor heterogeneity and could therefore provide an early treatment window allowing individualized therapeutic interventions.

Dysfunction of the basal ganglia plays a key role in the development of motor and non-motor symptoms in PD [15]. More specifically, depletion of dopamine in the putamen has been correlated with rigidity and bradykinesia while dopaminergic depletion in the caudate was associated with executive dysfunction [16]. The higher incidence of these symptoms in the PIGD compared to the TD subtype [5, 17] corresponds with differential striatal dopamine uptake patterns [18–20]. In agreement with these results, a previous study of our group found decreased functional connectivity (FC) between the caudate and putamen which correlated with motor and cognitive impairment using resting-state functional magnetic resonance imaging (rs-fMRI). Instead, a specific hyper-connectivity pattern between motor cortical areas and the inferior parietal lobule suggested a compensatory mechanism in TD. Of all the subtype-specific FC alterations at the whole-brain level, 65% were hypo-connections and 35% were hyper-connectivity in PIGD compared to TD [21].

Previous structural imaging studies showed decreased white matter integrity in the corpus callosum and superior longitudinal fasciculus in PIGD [12, 22]. However, other studies did not find evidence to support white matter changes in PD subtypes when directly comparing PIGD to TD [23], with the exception of widespread grey matter changes in the caudate and major cortical lobe areas [10]. Other relevant data come from diffusion imaging studies in the context of freezing of gait (FOG), a prominent symptom in PIGD, but not in TD. These studies found FOG-related decreases in microstructural integrity in tracts connecting the striatum with frontal, motor and sensory cortical areas [24], as well as altered characteristics in tracts originating in the pedunculopontine nucleus [25–27].

While FC is suggested to be at least partially related to structural connectivity [28], the structure-function relationship is currently not well understood. As local white matter deficits can lead to more elaborately distributed functional consequences [29], it is unclear if FC changes represent actual structural deficits or hemodynamic fluctuations. So far, no study investigated structural brain deficits in relation to FC changes in the context of PD subtypes.

In this study, we used three approaches to elucidate which white and grey matter alterations may underlie the differential FC patterns in PIGD and TD. First, we investigated whole brain differences in white matter integrity using Tract-Based Spatial Statistics (TBSS), which is a more robust method for detection of fractional anisotropy (FA) changes in comparison with voxel-wise techniques [30, 31]. We expected to find reduced white matter integrity in tracts between the striatum and cortical motor areas in PIGD compared to TD [24] as well as more widespread PIGD-specific FA reductions in addition to those found in the corpus callosum and the superior longitudinal fasciculus [12, 22, 24].

Second, we used probabilistic tractography to map white matter tracts between regions of interest that showed subgroup-specific FC changes in our previous rs-fMRI analysis [21]. Within these tracts, we looked for differences in white matter integrity between subgroups comparable to those investigated in the rs-fRMI study and correlated the respective structural and functional connectivity measures.

Third, we also performed a shape and volume analysis of the putamen, caudate and pallidum because of their key roles in subtype pathophysiology [18, 19, 21, 32, 33]. Previous grey matter studies in PD subtypes already reported widespread atrophy in PIGD compared to TD [10], although shape and volume analyses of the basal ganglia were unable to detect differences [34, 35]. However, these studies had small sample sizes or used lower magnetic field strengths during image acquisition. In the current study, we were able to enhance the detection power for basal ganglia shape differences. We expected to find the largest alterations in the caudate, with reduced volumes in the PIGD subgroup [36–38].

## Methods

### Subjects

Sixty-eight PD patients and 19 healthy age-matched controls were included. Patients were classified as PIGD (n = 41), TD (n = 19) or indeterminate (n = 8) based on subscores of the MDS-UPDRS parts II and III [1, 2] while ‘off’ medication, i.e. at least 12 hours after last medication intake. Therefore, the ratio of the average TD-score (score on items 2.10, 3.15 (a-b), 3.16 (a-b), 3.17 (a-e) and 3.18 of the MDS-UPDRS divided by 11) and the average PIGD-score (score on items 2.12, 2.13, 3.10, 3.11 and 3.12 of the MDS-UPDRS divided by 5) was assessed. If this was higher than 1.15 patients were considered to be TD and if this was lower than 0.9, patients were considered to be PIGD [2]. Subjects with in-between scores were considered as indeterminate and were excluded from further analyses. Patients were included if diagnosed with PD according to the UK Brain Bank criteria. Exclusion criteria were Mini-Mental State Examination (MMSE)-score <24 and presence of neurological comorbidities or contra-indications for MRI. Disease severity was assessed using the MDS-UPDRS part III and Hoehn and Yahr (H&Y) staging while ‘off’ medication. Patients were screened for Mild Cognitive Impairment (MCI) using level 1 MDS-criteria [39, 40] (i.e. a MoCA score < 26). The study was approved by the local ethics committee of the University Hospitals Leuven and all patients gave written informed consent prior to participation according to the Declaration of Helsinki.

### Image acquisition

All subjects underwent a Diffusion Tensor Imaging (DTI) MRI scan while “off” medication (Philips 3T ACHIEVA MRI scanner (Best, The Netherlands)) using a diffusion-weighted spin-echo sequence (duration: 633s, slice number: 58; slice thickness: 2.5mm; repetition time (TR): 7600ms; echo time (TE): 65ms; flip angle: 90°; matrix: 96x94; FOV: 240x200x145mm). The implemented b-value was 1300s/mm^2^ applied in 61 uniformly distributed directions and 1 image (58 slices) without diffusion weighting (B0) was obtained. In addition, a high-resolution anatomical T1-weighted sequence was acquired (T1 Turbo Field Echo (TFE) sequence, duration: 383ms; slice number: 182; slice thickness: 1.2mm; TR: 9.624ms; TE: 4.6ms; flip angle: 8°; matrix: 256x256; FOV: 21x250x250mm).

### DTI pre-processing

Diffusion data were visually screened for artifacts and signal dropout in FSLview (version 4.0.1). Four (1 PIGD and 3 TD) patients were excluded due to large image artifacts and 1 PIGD patient was excluded due to an excessive amount of signal dropout, bringing the final sample size to 39 PIGD, 16 TD and 19 controls. FMRIB Sofware Library (FSL) version 5.0.0 was used for data pre-processing (for details see the supporting information). Fractional anisotropy (FA) and mean diffusivity (MD) were calculated. Decreased FA and increased MD are indicative of worse microstructural white matter integrity.

### Shape analysis pre-processing

T1 anatomical images were registered and segmented using the FIRST tool implemented in FSL v5.0.0 [41]. Registration and segmentation results were visually reviewed and led to exclusion of 1 PIGD patient due to faulty segmentation. Total intracranial volume was calculated as the sum of white matter, grey matter and cerebrospinal fluid volumes using the segmentation tool implemented in SPM8.

### Tract-based Spatial Statistics

Tract-based Spatial Statistics (TBSS) were used to investigate white matter integrity at the whole-brain level in a voxel-wise manner [42]. Each subjects’ FA images were first realigned to a standard FA template (FMRIBFR_1mm) and subsequently to the FA image of the most representative subject, i.e. the subject to which all other subjects’ realignment was minimalized. Realigned images were then registered to MNI space and averaged over all subjects to create a mean FA image, which was ‘reduced’ to a group-averaged FA skeleton, thresholded at FA > 0.2. Next, each subjects’ FA image was projected onto this skeleton and the FA warps and skeleton projections were applied to the MD images.

### Probabilistic tractography

Subcortical and cortical regions of interest (ROIs) were selected based on a previous rs-fMRI study, which was performed in the same cohort [21]. In a previous study, we found significant functional connectivity alterations between areas of the fronto-parietal and motor control network in PIGD compared to TD patients [21]. These regions of interest pairs were used as seed and target regions for the probabilistic tractography (see S1 Table). The BEDPOSTX diffusion model was applied to calculate probabilistic distributions of the diffusion parameters in each voxel and to model crossing fibers [43] (for details see S1 File). The resulting tracts were used as a mask to extract mean FA and MD values for each subject’s skeletonized image generated during TBSS analysis. Correlations between PIGD and TD-scores and subgroup-specific FA and MD alterations in the tracts were tested using age and Levodopa Equivalent Dose (LED)-corrected partial correlations in the whole patient group (α was set at 0.05).

### Structure-function relationship

The correlation between functional and structural connectivity was calculated using partial correlation with age and LED as covariates in the entire patient group. The Fischer Z transformed Pearson correlation coefficients between ROIs were used as functional connectivity measures and the mean FA and MD values of the tracts connecting the same ROI’s were used as structural connectivity measures.

### Shape analysis

The registered and segmented T1 images were used to perform a volumetric and shape analysis of the bilateral caudate, putamen and pallidum. Shapes were calculated using the vertex analysis implemented in FSL v.5.0.0 (for details see S1 File).

### Statistics

Subject characteristics between groups were compared using independent T-tests or Mann-Whitney U tests for continuous variables (depending on normality of the data) and Chi-squared tests for categorical variables. Mean FA and MD values from the identified tracts and total volumes of the bilateral caudate, putamen and pallidum were compared between subgroups using ANCOVA’s with age and LED as covariates. LED was included as covariate as it was significantly different between patient subgroups and might be a marker for disease severity. Between group differences for the shape analysis and TBSS results were calculated using a General Linear Model. The design matrix consisted of T-contrasts with age and LED as covariates for comparison between patient subgroups. Voxel-wise statistical analyses were performed using 5000 randomized permutations. Threshold-Free Cluster Enhancement (TFCE) p-value images were considered at p<0.05. Due to the high inter-dependency of the FA and MD measures in the identified tracts (see S2 Table) and the exploratory nature of this study, we did not apply Bonferroni corrections for multiple testing.

## Results

### Subject characteristics

Table 1 shows subject characteristics of patient subgroups and healthy controls. Age did not significantly differ between subgroups and controls, but the PIGD group was slightly older than the TD group and had higher LED-values. To account for these difference, all further analyses were performed with age and LED as covariates. In addition MMSE and MoCA-scores were lower in PIGD compared to controls. There were no significant differences between groups for sex, disease dominance, MCI prevalence, disease duration and disease severity (MDS-UDPRS III and H&Y stage).

**Table 1:** Subject characteristics.

	PIGD (n = 39)	TD (n = 16)	CTRL (n = 19)	p-value		
**Age (years)**	62.4 (±11.0)	55.1 (±7.8)	58.1 (±8.9)	0.02	0.14	0.30
**Sex (M/F)**	24/15	9/7	14/5	0.72	0.36	0.28
**Disease duration (years)**	6.9 (±4.4)	4.8 (±1.7)	NA	0.06		
**MDS-UPDRS III (0–132)**	29.4 (±13.1)	28.9 (±10.3)	NA	0.88		
**H&Y I (%)**	5.1	18.7	NA	0.23**		
**H&Y II (%)**	71.8	68.8	NA			
**H&Y III (%)**	23.1	12.5	NA			
Disease dominance (L/R)*	15/23	4/11	NA	0.53		
**LED (mg/day)**	525.7 (±233.9)	249.2 (±189.8)	NA	<0.001		
**MMSE (0–30)**	28.1 (±1.8)	28.9 (±1.4)	29.3 (±0.8)	0.10	0.01	0.55
**MoCA (0–30)**	25.3 (±3.5)	26.9 (±2.9)	27.5 (±1.6)	0.12	0.002	0.41
**MCI (Y/N)**	17/22	3/13	NA	0.12		
**PIGD-score (0–20)**	4.3 (±2.8)	1.7 (±1.1)	NA	<0.001		
**TD-score (0–44)**	3.0 (±2.5)	9.3 (±3.0)	NA	<0.001		
**Total intracranial volume (cm**^**3**^**)**	1875.7 (±282.3)	1844.9 (±167.0)	1840.8 (±135.9)	0.69	0.61	0.94

Means and standard deviations (SD) are reported.

*Two patients did not present with a clear disease dominant side.

**P-value indicates comparison of proportions of each H&Y stage between groups.

P-values indicate comparison between PIGD and TD. If more than one p-value is reported, the first column represents the PIGD-TD comparison, the second column represents the PIGD-CTRL comparison and the third column represents the TD-CTRL comparison. CTRL: controls; H&Y: Hoehn and Yahr; LED: Levodopa Equivalent Dose; MDS-UPDRS: Movement Disorders Society Unified Parkinson’s Disease Rating Scale; MCI: Mild Cognitive Impairment; MMSE: Mini-Mental State Examination; MoCA: Montreal Cognitive Assessment; NA: Not applicable; PIGD: Postural Instability and Gait Disorder; TD: Tremor Dominant

### TBSS

**Fig 1:**
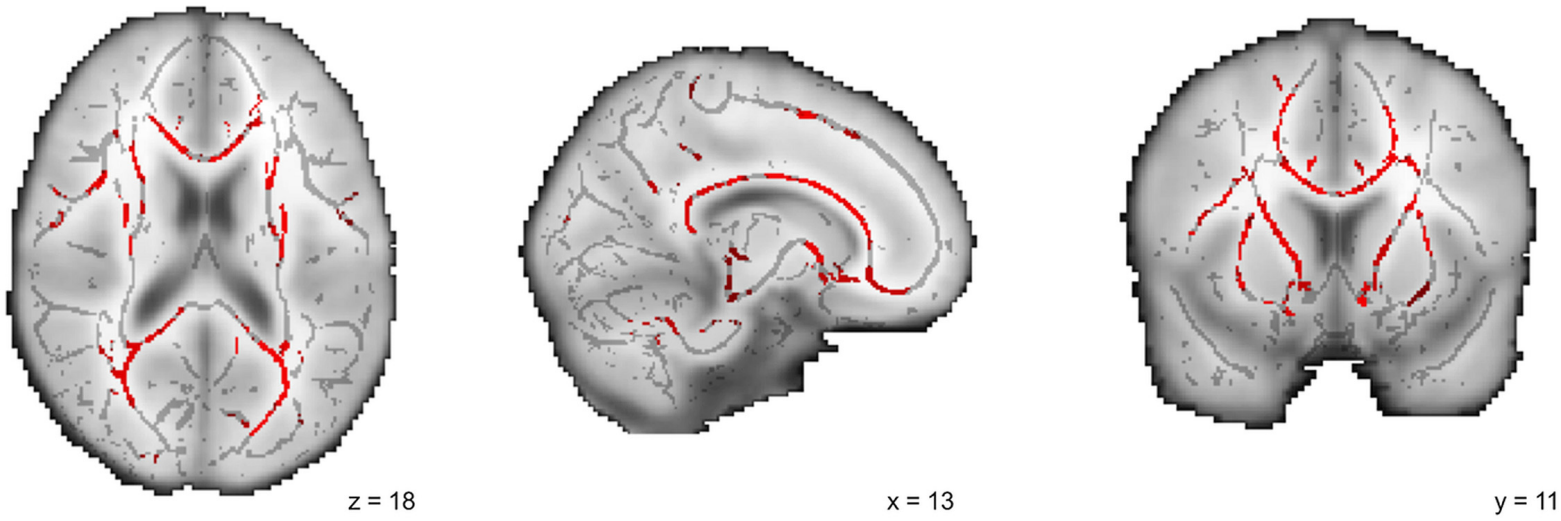
TBSS results for PIGD patients compared to controls. Red clusters represent white matter areas with significantly (TFCE-corrected p < 0.05) decreased FA in PIGD patients compared to controls. No such areas were found for TD compared to controls. One representative coronal, sagittal and axial slice are shown with the TBSS results overlain on the mean FA skeleton (in grey).

Direct comparison of both patient groups did not reveal any differences in FA or MD at the whole-brain level. However, PIGD showed significantly decreased FA compared to controls in the splenium, body and genu of the corpus callosum, in the anterior corona radiata, the bilateral inferior fronto-occipital fasciculi, the bilateral anterior thalamic radiations, the bilateral cingulate gyri, the bilateral corticospinal tracts and the bilateral superior longitudinal fasciculi (Fig 1). In contrast, comparison of TD with healthy controls showed no differences in FA or MD.

### Probabilistic tractography

**Fig 2:**
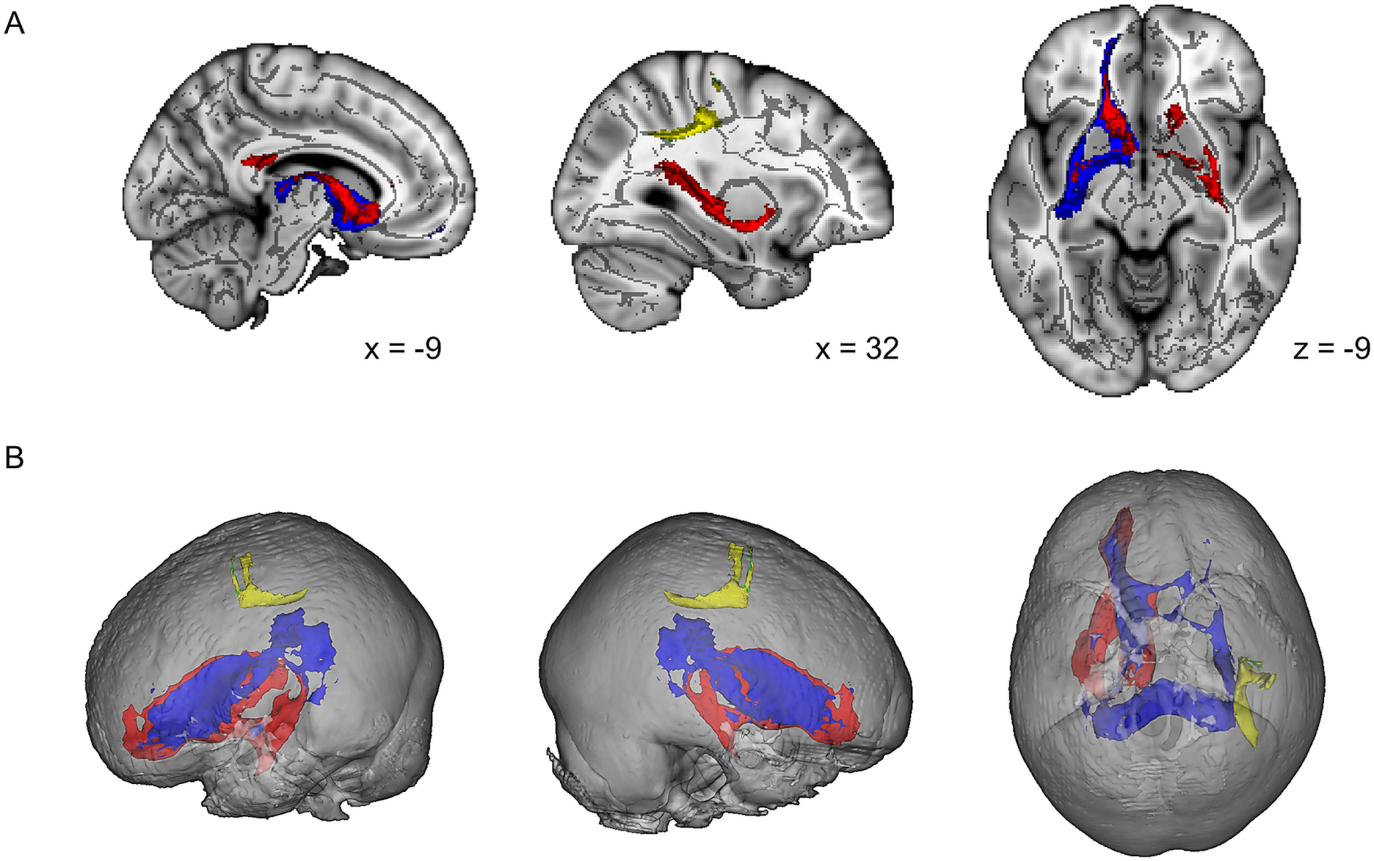
Altered white matter tracts in patient subgroups compared to controls. Tracts identified with probabilistic tractography having increased MD in patient subgroups compared to controls are shown. The red tract connects the left caudate with the contralateral ventral putamen. The blue tract connects the left caudate with the ipsilateral ventral putamen. Both tracts showed increased MD in both patient subgroups compared to controls. The yellow tract connects the right inferior parietal lobule with the right M1 and the green tract connects the right inferior parietal lobule with the right premotor cortex. Both tracts showed increased MD in TD compared to PIGD and controls. Part A shows two sagittal and one axial brain slices where the identified tracts are overlain on the mean FA skeleton. As the yellow and green tract overlap for a large part, only the yellow tract is visible. Part B shows glass brains with 3D representations of the identified tracts.

Table 2 reports the tracts that were identified in the probabilistic tractography based on the seed and target regions that showed altered functional connectivity in PD subtypes in our previous study [21]. Fig 2 shows those tracts that had significantly different MD values between subgroups. Direct comparison of the PIGD and TD subgroup showed increased MD in tracts connecting the right premotor cortex/M1 and right inferior parietal lobule in TD compared to PIGD. In line with these results, TD had increased MD compared to controls in the same tracts. In contrast, both TD and PIGD showed higher MD-values compared to controls in the tracts connecting the left caudate and the bilateral ventral putamen. There were no differences between groups for FA.

**Table 2:** FA and MD alterations in white matter tracts between PIGD, TD and controls.

Tract origin	Tract destination	PIGD		TD		CTRL		PIGD-TD p-value		PIGD-CTRL p-value		TD-CTRL p-value	
		FA x E-01	MD x E -04 mm^2^.s^-1^	FA x E-01	MD x E -04 mm^2^.s^-1^	FA x E-01	MD x E -04 mm^2^.s^-1^	FA	MD	FA	MD	FA	MD
Left caudate	Left premotor cortex	6.05 (±0.36)	6.42 (±0.29)	6.07 (±0.32)	6.41 (±0.21)	6.14 (±0.32)	6.35 (±0.25)	0.96	0.20	0.31	0.71	0.68	0.22
Left caudate	Left dorsal putamen	4.44 (±0.20)	7.04 (±0.35)	4.46 (±0.21)	6.93 (±0.31)	4.50 (±0.15)	6.83 (±0.21)	0.98	0.39	0.36	0.08	0.59	0.06
Left caudate	Left ventral putamen	4.05 (±0.17)	7.36 (±0.31)	4.08 (±0.25)	7.25 (±0.29)	4.12 (±0.14)	7.12 (±0.19)	0.64	0.44	0.26	**0.01**	0.46	**0.02**
Left caudate	Right dorsal putamen	5.04 (±0.26)	7.40 (±0.75)	5.09 (±2.45)	7.13 (±0.31)	5.09 (±0.15)	7.09 (±0.23)	0.72	0.91	0.61	0.22	0.88	0.27
Left caudate	Right ventral putamen	5.22 (±0.20)	7.39 (±0.37)	5.26 (±0.24)	7.26 (±0.29)	5.33 (±0.20)	7.11 (±0.29)	0.70	0.36	0.13	**0.02**	0.38	**0.04**
Left dorsolateral prefrontal cortex	Right middle frontal gyrus	5.41 (±0.55)	6.84 (±0.79)	5.50 (±0.48)	6.73 (±0.63)	5.64 (±0.41)	6.44 (±0.58)	0.55	0.16	0.25	0.15	0.35	0.10
Right caudate	Right dorsal putamen	4.47 (±0.20)	7.00 (±0.37)	4.50 (±0.19)	6.88 (±0.29)	4.50 (±0.18)	6.81 (±0.24)	0.87	0.58	0.73	0.16	0.87	0.13
Right primary motor cortex	Right inferior parietal lobule	4.25 (±0.35)	6.71 (±0.26)	4.53 (±0.32)	6.74 (±0.30)	4.46 (±0.25)	6.57 (±0.25)	0.40	**0.02**	0.38	0.17	0.53	**0.03**
Right premotor cortex	Right inferior parietal lobule	4.22 (±0.32)	6.78 (±0.26)	4.36 (±0.30)	6.79 (±0.31)	4.34 (±0.24)	6.62 (±0.26)	0.63	**0.02**	0.31	0.12	0.86	**0.02**

Means and standard deviations are shown for the FA and MD values that were extracted from the identified tracts using probabilistic tractography. Between-group analyses were performed using age as covariates. For the comparison between patient subgroups, LED and age were included as covariates. CTRL: controls; FA: Fractional Anisotropy; MD: Mean Diffusivity; PIGD: Postural Instability and Gait Disorder; TD: Tremor Dominant.

Higher TD-scores correlated to increased MD in the tracts connecting the right inferior parietal lobule with the right premotor cortex (R = 0.28; p = 0.04) and the right primary motor cortex (R = 0.28; p = 0.04). There were no significant correlations with PIGD- and TD-scores, nor between FC and structural connectivity measures (R: 0.03–0.25).

### Volume and shape analysis

**Fig 3:**
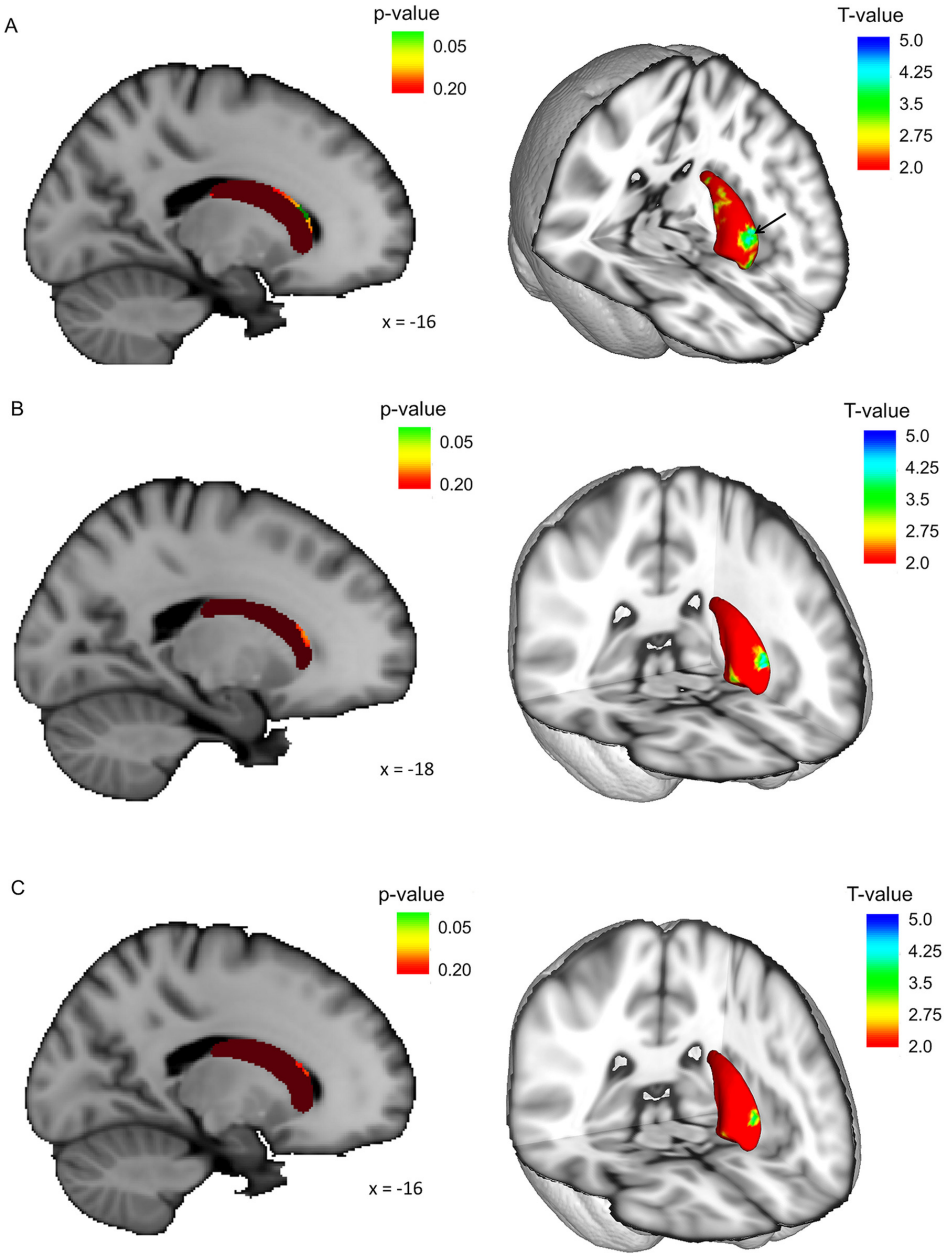
Shape deformations in the left caudate nucleus. All figures on the left show the left caudate that is color-coded according to p-values. Dark red colors represent p-values above 0.2. Lighter red, orange and yellow represent lower p-values and green colors indicate p-values below 0.95. All figures on the right show a 3D representation of the left caudate with color-coding for the T-values. (A) shows the results of the PIGD-CTRL comparison. (B) shows the results of the PIGD-TD comparison with age correction. (C) shows the results of the PIGD-TD comparison with age and LED correction.

Comparison of total volumes of the caudate, putamen and pallidum between groups did not reveal any differences. In contrast, the shape of the left caudate was altered when comparing PIGD to controls and not in TD compared to controls. Vertex analysis showed a significantly decreased volume in the curvature dorsal to the left caudal head with age and LED correction in PIGD compared to controls (p < 0.05) (Fig 3A). Although not significant, the same regional changes were found in PIGD compared to TD when controlled only for age, but not for LED (p = 0.067)(Fig 3B). Adding LED as a covariate to the PIGD-TD analysis led to further increase of the p-value to 0.2 (Fig 3C). No shape alterations between groups were found for the right caudate and the bilateral putamen and pallidum.

## Discussion

This study aimed to increase insight in the neural alterations underpinning PD motor subtypes. Structural and functional information was combined by using FC results from a previous study [21] as a framework to investigate the underlying structural brain differences in PIGD compared to TD. Therefore, this study was the first to interpret multimodal imaging techniques as applied to motor heterogeneity in PD.

The current study revealed globally decreased FA in several major white matter tracts suggesting widespread microstructural decline in PIGD versus controls corroborating the global pattern of functional hypo-connectivity in our rs-fMRI study [21]. This is also in line with previously reported brain-wide structural deficits in PIGD compared to TD showing a higher burden of white matter intensities [9] and increased grey matter atrophy [10]. Moreover, our results confirm earlier DTI work in PD subtypes that showed decreased FA in the bilateral superior longitudinal fasciculi, bilateral anterior corona radiata, and corpus callosum [12, 22]. The superior longitudinal fasciculi are vast white matter tracts connecting all the major cortical lobes in the brain transmitting information through cortico-cortical and cortico-pontine-cerebellar circuits [12, 44]. Damage to this structure was associated with bradykinesia in de novo PD patients [45], freezing of gait [24] as well as with visuospatial processing and attention to goal-directed behavior due to its role in the dorsal and ventral fronto-parietal network [46, 47]. Similarly, the corpus callosum contains crossing fibers that project to major cortical areas that are related to motor and sensory functions [48]. Particularly abnormalities in the posterior region of the body and splenium of the corpus callosum were associated with gait dysfunction [22]. The PIGD-specific pattern of microstructural decline thus provides a framework for the development of PIGD-related motor and non-motor symptoms [49]. In addition, FA was also reduced in PIGD compared to controls in the bilateral inferior fronto-occipital fasciculi, anterior thalamic radiations, angular gyri and corticospinal tract. A similar pattern of white matter disintegration was reported earlier in the context of FOG [24, 26] and supports the hypothesis of FOG being a subgroup within PIGD [50]. Yet, we did not find altered white matter integrity in cerebellar and pedunculopontine tracts which were repeatedly linked to FOG pathophysiology [24, 25, 27]. Overall, we found less pronounced WM changes when comparing PIGD and TD directly compared to our previous FC study [21], confirming that the structure-function relationships are complex to interpret.

Previously, the decreased FC found in PIGD was understood as increased neural deterioration which correlated with worse motor and cognitive outcomes. Following the same rationale, TD-specific increased FC was interpreted as a compensatory mechanism as it was correlated to improved motor outcomes. The current results showed that part of the FC differences found between PIGD and TD corresponded with structural alterations. We found increased MD, indicative of reduced microstructural organization, in the tracts connecting the left caudate with the bilateral ventral putamen in both patient groups compared to controls. Decreased intra-striatal resting-state FC has been shown to be specific to the ‘off’ state in PD, leading to impaired integration of parallel cortico-striatal circuits and decoupling of thalamic and sensorimotor networks [51]. Decoupling of these networks was accompanied by increased coupling of the striatum with the fronto-parietal network, suggesting a compensatory cognitive control mechanism [51]. Altered connectivity between the striatum and fronto-parietal areas has also been strongly related to the characteristic defective motor automaticity in PD [52]. Specifically connectivity with the posterior putamen was associated with difficulties in shifting acquired motor skills to the automatic stage [53]. This mechanism was suggested to contribute to several motor symptoms in PD such as akinesia, FOG, decreased stride length, reduced arm swing, micrographia and abnormalities in facial movements [52].

In contrast with our FC results, we found increased MD (indicating worse white matter integrity) in the tracts connecting the right inferior parietal lobule with the ipsilateral premotor cortex and M1 in TD compared to PIGD and controls. This coincided with increased FC between these regions, which we earlier interpreted to be a TD-specific compensatory mechanism due to correlations with better motor outcomes and lower PIGD-scores [21]. The present structural connectivity results, however, prompt us to refine or revise this interpretation. The seemingly contradictory findings could be explained in three ways. First, the TD-specific loss of white matter structural integrity may refute the idea of a compensatory pathway and instead suggest a TD-specific deficit. In line with this hypothesis, the loss in white matter integrity may lead to a reduction in an inhibitory signaling pathway possibly explaining the increased functional connectivity.

Second, the notion of compensatory activity in this area may still hold through the involvement of a region that is structurally connected to both the inferior parietal lobule and the premotor cortex and M1 and which mediates the FC increases between these cortical areas [54]. Supported by the correlation with TD-scores, the current results strongly suggest a primary structural deficit between the inferior parietal lobule and the premotor cortex and M1 in TD compared to controls. This does not seem to result in a functional deficit, but may rather lead to increased FC by over-compensation through an alternative pathway. Previous DTI studies have established connecting fibers between the rostral part of the inferior parietal lobule and the ventral part of the premotor cortex, coinciding with the superior longitudinal fasciculus [55, 56]. At a functional level, increased connectivity between these areas has been associated with better motor learning capacities in older adults [57], where specifically the right inferior parietal lobule is involved in attention and motor preparation and is a key hub within the ventral attention network [58]. The increased FC may thus be mediated by the ventral attention network, highlighting the middle and inferior frontal regions as possible candidates for this function as they are also structurally connected to the inferior parietal lobule [58]. This notion may also explain why some of the FC changes were not associated with altered white matter integrity [29]. Alternatively, the relatively small ROI sizes in large cortical areas may have restricted the tractography procedure spatially in cortical and cerebellar areas such as in the posterior cingulate cortex, part VI of the cerebellum and the cerebellar vermis. Future work will benefit from using masks that cover larger areas instead of focusing on functional hotspots.

Third, methodological pitfalls may explain the results. Multi-modal neuroimaging studies have suggested that a moderately strong correlation exists between functional and structural connectivity measures implying that increased FC alterations are co-existent with better axonal integrity of connecting fibers [59, 60]. Although generally FC and DTI findings correspond, in some cases overlap may not apply because resting-state FC can arise from mechanisms other than direct structural connectivity [61]. In this regard, a recent review concluded that increased structural connectivity predicts increased FC, but not vice versa [28]. In addition to the previously mentioned mediation by a third region, altered FC in the absence of structural connectivity changes may also be explained by common input from other brain areas [62]. This may be a particularly valid explanation for the lack of correlations between functional and structural connectivity strengths in the tracts connecting different parts of the striatum, in particular in the context of the presence of PD pathology when complex compensatory networks are at play [15].

In addition, limitations inherent to the imaging technique are a potential source for confounding. Although the PROBTRACKX model takes crossing fibers into account, detection of relatively small fiber bundles perpendicular of major tracts still imposes a technical challenge [61]. Moreover, probabilistic tractography leads to erroneous results when fiber tracts are severely damaged. FA values in damaged regions are very low and are thus excluded from the tract leading to an underestimation of the microstructural damage [63].

This study also probed grey matter alterations of the major nuclei of the basal ganglia, as previous studies suggested their involvement in pathophysiology of motor subtypes in PD [18, 19, 32, 33]. We found that the rostrodorsal part of the left caudate head showed more atrophy in PIGD compared to controls. Loss of dopaminergic neurons that project to this specific part of the caudate has been associated with worse neuropsychological outcome measures and increased risk of dementia in PD [64–66]. It has been shown that cognitive control mechanisms are affected in PIGD. Previous studies found greater executive impairment compared to TD patients [4, 6], while imaging studies have shown PIGD-specific decreased activity in the default mode network [11]. This suggests that defective cognitive control mechanisms might contribute to PIGD pathophysiology. However, this structural deficit may not have permeated yet to the functional or behavioral level as we found limited cognitive impairment in PIGD compared to TD. PIGD did show lower MMSE and MoCA scores compared to controls, which was not the case for TD patients. It is possible that the executive dysfunction in PIGD was not severe enough to be directly detected by our cognitive tests, but instead largely presented as alterations in motor performance [8] mediated by altered cognitive control.

In contrast to the current results, previous morphological studies in PD subtypes were unable to show any differences in subcortical structure shapes [34, 35]. Improved statistical power due to larger sample sizes in the current study possibly explains this discrepancy. Atrophy of the caudate head correlated with scores on the Montreal Cognitive Assessment (MoCA) in early PD patients [67] and with cognitive decline in a study comparing non-demented PD patients with PD patients with Mild Cognitive Impairment (MCI) and PD with dementia [68]. The results of the direct comparison between PIGD and TD were modulated by adding LED as a covariate, which reduced the strength of the results. Given that our patients were tested ‘off’ medication, this confounding impact was surprising. One possible explanation could be that including an additional covariate reduced the statistical power of the model, rendering less significant results. However, it is also possible that higher LED doses have a protective effect on grey matter loss, the mechanism of which needs to be elucidated by future studies.

Although we analyzed white matter tracts between the left caudate and some cortical areas, we did not include regions of the associative loop such as the dorsolateral prefrontal cortex, the frontal pole and the pre-supplementary motor area [65, 66, 69]. Therefore, future tractography studies are warranted to visualize the fiber pattern converging onto the caudal head and elucidate the differential fiber properties in PD subtypes. This will be a crucial step in establishing the contribution of the altered dynamic interplay between pure motor control and cognitive modulation to subtype pathophysiology.

The fact that our PIGD cohort was older than our TD cohort is a limitation of this study. Rather than applying post-hoc matching procedures, we opted to increase statistical power by maximizing the sample size and correcting age-related effects by incorporating this as a covariate of no interest in all analyses. Due to the exploratory nature of this study and strong inter-dependency of the FA and MD outcome measures, we did not to apply multiple comparison corrections, which needs to be taken into account when interpreting our tractography results. Although our PIGD and TD cohorts had comparable disease severities and all analyses were stringently corrected for LED, effects of disease progression cannot be excluded. Longitudinal studies in independent patient cohorts are recommended to make definite conclusions on how white matter burden is involved in determining PD phenotypes and its progression and should incorporate Fluid Attenuated Inversion Recovery images to allow pinpointing the underlying reasons for microstructural white matter deficiencies. As the distribution of disease dominance was not significantly different between patient subgroups and two patients had symmetrical disease characteristics, we did not flip the images according to the disease-dominant brain side. This may have underestimated subtype differences. Finally, although our results showed altered functional and structural connectivity between fronto-parietal and motor control networks in similar patient cohorts, they were unable to fully clarify the relationship between structure, function and behavior. Measures of effective connectivity, which quantify the influence that one neural system exerts over another, may provide a useful tool in elucidating this matter in future studies [70].

## Conclusion

This study provides supportive evidence that FC changes related to PD motor subtypes are partly underpinned by microstructural deficits in white matter tracts. A TD-specific structural deficit was identified in the connecting fibers between the inferior parietal lobule and motor cortical areas, while PIGD-related deficits suggested impaired white matter tracts involved in cognitive and motor control. In addition, we found a deformation in the rostrodorsal part of the caudate head in PIGD compared to controls, which proved not present in TD. The decreased integrity in major white matter tracts found in PIGD, suggests a partly common mechanism with FOG. Hence, the present results provide a structural framework to explain symptomatic heterogeneity in PD.

## Supporting Information

S1 File Supporting information methods. (DOCX)

S1 Table ROI pairs for probabilistic tractography. (DOCX)

S2 Table Correlation matrix of mean FA and MD values within identified tracts. Pearson correlations coefficients are shown on the top row of each cell. The bottom row of each cell is the p-value of the correlation.
(DOCX)
