# Supplementary material for: Structural Brain Alterations in Motor Subtypes of Parkinson’s Disease: Evidence from Probabilistic Tractography and Shape Analysis
Source: PLoS One. 2016 Jun 17;11(6):e0157743. doi: 10.1371/journal.pone.0157743 (PMC4912098; doi:10.1371/journal.pone.0157743)
Supplement: S1 File — (DOCX) [file pone.0157743.s001.docx]

**Supporting Information**

**DTI pre-processing**

All images were corrected for subject motion and eddy-current distortions by affine registration of each diffusion weighted image to the mean of subject’s diffusion-weighted images, followed by rigid body registration of that to the B0 image. Masks were created to exclude regions with signal dropout from individual diffusion-weighted imaged at the slice level during the fitting of the diffusion tensor model.

**Probabilistic Tractography**

S1 Table shows all ROI-pairs that had significant or borderline-significant (p<0.08) functional connectivity (FC) alterations in the PIGD compared to TD subgroup and were selected for probabilistic tractography.

The signal dropout mask that was generated during pre-processing was also included in the model. Constrained probabilistic tractography was initiated from the ROIs shown in the first column of S1 Table towards the ROIs shown in the second column. For each voxel within seed ROI, 5000 samples of the principal diffusion directions with a curvature threshold of 0.2 were generated repetitively [1]. Samples that reached the target ROI were retained. For each subject, distributions of the diffusion trajectories were thresholded at 0.02% of the maximum value in the connectivity map to remove noise. Next, these maps were transformed into Montreal Neurological Institute (MNI) space using combined affine and nonlinear registrations, binarized and summed across subjects to determine the most probable spatial trajectory of each tract [2]. Voxels that were part of a tract in >85% of all subjects’ maps were retained [3].

**Shape analysis**

This method is based on a deformable mesh model of subcortical structure shapes constructed with information from parametrization of surface vertices. Construction of this learning model was based on manually-labeled training data of 336 normal and pathological brains [4]. The normalized intensities of each subject’s T1 image along the surface of the structure were sampled and modeled using the FIRST tool, which subsequently searched through different combinations of modes of variations to find the most probable shape given the sampled intensities.

**Correlation matrix of mean FA and MD values within identified tracts**

Mean FA and MD values within all tracts that were identified using probabilistic tractography were Pearson correlated within the entire cohort (S2 Table).

**References**

1. Colloby SJ, Firbank MJ, Thomas AJ, Vasudev A, Parry SW, O'Brien JT: **White matter changes in late-life depression: a diffusion tensor imaging study**. *J Affect Disord* 2011, **135**(1-3):216-220.

2. Aron AR, Behrens TE, Smith S, Frank MJ, Poldrack RA: **Triangulating a cognitive control network using diffusion-weighted magnetic resonance imaging (MRI) and functional MRI**. *J Neurosci* 2007, **27**(14):3743-3752.

3. Muthusamy KA, Aravamuthan BR, Kringelbach ML, Jenkinson N, Voets NL, Johansen-Berg H, Stein JF, Aziz TZ: **Connectivity of the human pedunculopontine nucleus region and diffusion tensor imaging in surgical targeting**. *J Neurosurg* 2007, **107**(4):814-820.

4. Patenaude B, Smith SM, Kennedy DN, Jenkinson M: **A Bayesian model of shape and appearance for subcortical brain segmentation**. *Neuroimage* 2011, **56**(3):907-922.
