# Supplementary material for: Structural Brain Alterations in Motor Subtypes of Parkinson’s Disease: Evidence from Probabilistic Tractography and Shape Analysis
Source: PLoS One. 2016 Jun 17;11(6):e0157743. doi: 10.1371/journal.pone.0157743 (PMC4912098; doi:10.1371/journal.pone.0157743)
Supplement: S1 Table — (DOCX) [file pone.0157743.s002.docx]

**S1 Table. ROI pairs for probabilistic tractography**

| **ROI 1** | **ROI 2** |
| --- | --- |
| Left caudate | Right ventral putamen |
| Left caudate | Left ventral putamen |
| Left caudate | Right dorsal putamen |
| Left caudate | Left dorsal putamen |
| Left caudate | Left pallidum |
| Left caudate | Left premotor cortex (PMC) |
| Left caudate | Right superior temporal lobe |
| Right caudate | Right dorsal putamen |
| Right dorsal putamen | Right cerebellum (lobule VI) |
| Right cerebellum (lobule VI) | Right inferior parietal lobule (IPL) |
| Right premotor cortex | Right inferior parietal lobule |
| Right primary motor cortex (M1) | Right inferior parietal lobule |
| Cerebellar vermis | Left posterior cingulate cortex |
| Cerebellar vermis | Right posterior cingulate cortex |
| Left dorsolateral prefrontal cortex (DLPFC) | Right middle frontal gyrus (MFG) |
