# Supplementary material for: Structural Brain Alterations in Motor Subtypes of Parkinson’s Disease: Evidence from Probabilistic Tractography and Shape Analysis
Source: PLoS One. 2016 Jun 17;11(6):e0157743. doi: 10.1371/journal.pone.0157743 (PMC4912098; doi:10.1371/journal.pone.0157743)
Supplement: S2 Table — Pearson correlations coefficients are shown on the top row of each cell. The bottom row of each cell is the p-value of the correlation. (DOCX) [file pone.0157743.s003.docx]

**S2 Table. Correlation matrix of mean FA and MD values within identified tracts**

| Correlations between MD in tracts  (R and p-value) | L caudate – L PMC | L caudate – L dorsal putamen | L caudate – L ventral putamen | L caudate – R dorsal putamen | L caudate – R ventral putamen | L DLPFC - R MFG | R caudate – R dorsal putamen | R M1 – R IPL | R PMC – R IPL |
| --- | --- | --- | --- | --- | --- | --- | --- | --- | --- |
| L caudate – L PMC | 1 | 0.81  <0.001 | 0.71  <0.001 | 0.58  <0.001 | 0.65  <0.001 | 0.57  <0.001 | 0.78  <0.001 | 0.53  <0.001 | 0.53  <0.001 |
| L caudate – L dorsal putamen | 0.81  < 0.001 | 1 | 0.94  <0.001 | 0.85  <0.001 | 0.87  <0.001 | 0.64  <0.001 | 0.92  <0.001 | 0.63  <0.001 | 0.67  <0.001 |
| L caudate – L ventral putamen | 0.71  <0.001 | 0.94  <0.001 | 1 | 0.78  <0.001 | 0.88  <0.001 | 0.53  <0.001 | 0.86  <0.001 | 0.60  <0.001 | 0.66  <0.001 |
| L caudate – R dorsal putamen | 0.58  <0.001 | 0.85  <0.001 | 0.78  <0.001 | 1 | 0.75  <0.001 | 0.54  <0.001 | 0.80  <0.001 | 0.54  <0.001 | 0.54  <0.001 |
| L caudate – R ventral putamen | 0.65  <0.001 | 0.87  <0.001 | 0.88  <0.001 | 0.75  <0.001 | 1 | 0.63  <0.001 | 0.83  <0.001 | 0.66  <0.001 | 0.76  <0.001 |
| L DLPFC - R MFG | 0.57  <0.001 | 0.64  <0.001 | 0.53  <0.001 | 0.54  <0.001 | 0.63  <0.001 | 1 | 0.58  <0.001 | 0.46  <0.001 | 0.51  <0.001 |
| R caudate – R dorsal putamen | 0.78  <0.001 | 0.92  <0.001 | 0.86  <0.001 | 0.80  <0.001 | 0.83  <0.001 | 0.58  <0.001 | 1 | 0.69  <0.001 | 0.72  <0.001 |
| R M1 –R IPL | 0.53  <0.001 | 0.63  <0.001 | 0.60  <0.001 | 0.54  <0.001 | 0.66  <0.001 | 0.46  <0.001 | 0.69  <0.001 | 1 | 0.90  <0.001 |
| R PMC – R IPL | 0.53  <0.001 | 0.67  <0.001 | 0.66  <0.001 | 0.54  <0.001 | 0.76  <0.001 | 0.51  <0.001 | 0.72  <0.001 | 0.91  <0.001 | 1 |

Pearson correlations coefficients are shown on the top row of each cell. The bottom row of each cell is the p-value of the correlation.
